# Glass Fillings, Jacket Crowns, Amalgam, Gold Caps, Bridges, Etc.

**Published:** 1895-06

**Authors:** William H. Trueman

**Affiliations:** Philadelphia


### GLASS FILLINGS, JACKET CROWNS, AMALGAM, GOLD CAPS, BRIDGES, ETC.—A SCRAP OF THEIR HISTORY.

BY WILLIAM H. TRUEMAN, PHILADELPHIA.

"In cases where a cavity is in front of a cutting tooth, the amalgam stopping is objectionable from its metallic appearance; but, if a small piece of thin platina be cut so as to fit the mouth of this cavity, and on each side of this a few catching points be soldered, glass, the color of the teeth, may be fused on one side, and the cavity being partly stopped with amalgam, the catching points on the side uncovered by the glass, are pressed into the amalgam firmly, in the course of an hour or two the glazed platina becomes fixed by the hardening of the amalgam; this operation, if neatly performed, must give the greatest satisfaction to the patient. It is two years since the idea of trying the experiment suggested itself to me, since then I have often practiced it, and I can say always satisfactorily.

"When teeth are very much decayed, discoloured, or have their enamel much injured or disfigured, caps of gold, platina, or palladium, may be stamped up to fit them with the greatest exactness; the fronts of these dental caps must be glazed, and they can then be worn with much benefit."

The above paragraphs are taken *verbatim et literatim* from a "Popular Treatise on the Structure, Diseases, and Treatment of the Human Teeth," by J. L. Murphy, published at London, England, in 1837, pages 200 and 201 (No. 1594, "Dental Bibliography," by C. Geo. Crowley). Of the glass used for this purpose he says, "Glass of any colour, may be bought in the cane ready for use. All dentists ought to be provided with some of various colours, as it is useful in many instances." He gives minute directions regarding the construction of suitable furnaces for baking porcelain teeth, and various formula for preparing bodies and enamel. On this point he says, "A good composition for teeth may be made of silex, two parts; potash, two parts; dry potter's clay, one part; these must be well beat up together so as to be perfectly mixed. They had better be put in a mortar, and having been beat up sometime, water may be added to form them into a paste; this, after being well ground in the mortar, is fit for use." Teeth made from this body were first baked, then colored, then coated as thickly as possible on the fronts and as thinly as possible on the backs with a mixture of one part silex and one and a half parts potash, carefully beat up with water into a paste the consistency of cream. After this had dried they were again placed in the oven and subjected to a fierce heat for about two or three hours, when "the potash and silex will be found to have melted into a good glaze."

This description, to those who are old enough, will recall the painted teeth of our old friend Samuel Stockton and his compeers; teeth that had to be held to the grindstone in a certain way to prevent the enamel surface chipping off; if in an unguarded moment this was neglected, the glaze was apt to chip, at times the entire face of the tooth would scale off, occasionally burying itself into the finger end and calling forth sundry unsaintly ejaculations.

So far as I now recall, the author quoted is the earliest writer I have met with who speaks approvingly of amalgam, or gives directions for its preparation.[1] While few acknowledged its use, we

[1] American Journal of the Dental Sciences, vol. ii., p. 155, September, 1841.

have ample evidence that it was used by many; and that at an early date, early in the forties, if not before, tin, platinum, gold, etc., were added to the silver used in dental amalgam. Of amalgam, on page 104, he says, "I have for many years, used an amalgam of silver, prepared in the following manner, and though there are objections against it, still, until something better is laid before the public, it will be found of a highly useful nature. The ingredients necessary for the cement are silver filings, thinnest silver leaf (to be had in small books), and quicksilver. A drop of quicksilver is poured out from the bottle into a small mortar, or if more convenient, on a cloth; on this is placed a leaf of silver from the book, which, being worked into the quicksilver, becomes speedily amalgamated with it, another leaf is then added, and so on until the amalgam is a thick paste; if there be too little quicksilver in this amalgam, the want will be made perceptible by its non-adhesion, and, on being worked between the fingers, by its crumbling. If, on the contrary, it possesses too much mercury, it will be too soft, and the mercury may easily be squeezed out."

"When the amalgam is brought to a proper consistency, having just sufficient mercury to enable it to be used as a paste, a small quantity of silver filings should be mixed with it: these absorb a portion of the quicksilver, and hence there is insufficient mercury left to keep the silver in a soft state: thus, after the filings are introduced, the amalgam gradually hardens. Here, then, we are in possession of a cement which may be used in a soft cold state; yet, on being placed in the tooth, speedily hardens in the cavity."

In "The Parent's Dental Guide; a Treatise on the Diseases of the Teeth and Gums, from Infancy to Old Age," etc., by William Imrie, Surgeon-Dentist, London, 1834 (No. 1583, Crowley's "Dental Bibliography"), I find on page 105 the following in relation to gold caps:

"When the back teeth have become shortened, and do not touch their opponents in the opposite jaw, one or more of them on each side of either jaw, as may be found most suitable and convenient, should be covered with *gold caps*."

"Indentations should be formed on the grinding surface of the caps to correspond with those of the teeth; and for this purpose they require to be raised on a brass model of the grinders, a process well known to dentists of ability and skill."

"When only a few molar teeth remain in the mouth, although they may be extensively decayed, it is essential to preserve them, for purposes of mastication, and also to prevent the front ones falling a sacrifice to undue pressure. For this purpose the decayed molars should be plugged and afterwards restored to their original dimensions by means of gold caps. This is an effectual way, and may be recommended to persons who have an antipathy to artificial teeth." In illustration he recites a case where "the whole of the double teeth on each side of the lower jaw" were covered with gold caps to relieve the upper front teeth of undue pressure and wear caused by a wearing down of the grinding surfaces of the molars. This case, so far as the cause, the described condition, treatment, and result is concerned, reads precisely as do many such cases related in the dental journals of the last few years.

William Imrie, in a foot-note, quotes from Patterson Clark. I find the words quoted in "A New System of Treating the Human Teeth," by J. Patterson Clark, M.A., Dentist." My copy is of the second edition, London, 1830 (No. 1574, Crowley's "Dental Bibliography"). The first edition was published in 1829. Mr. Clark, from page 162 to the end of the volume, page 199, gives many cases which resemble very closely the cap- and bridge-work of the present day. This portion of his book deserves a careful study by those interested in the history of this department of prosthetic dentistry; indeed, as the book may be inaccessible to many, a reprint of these pages might be of much interest. We have no hesitation in saying, after perusing them, that if the dental engine with its revolving tools and grindstones, and the various cements now in general use, had been known and in as general use when J. Patterson Clark wrote, the bridge-work of his time would, in all probability, have been as satisfactory as is that of to-day. The want of these modern helps undoubtedly caused it to become, for the time being, a lost art.

Throughout the book there is much a thoughtful dentist will read with keen interest if not with profit. Defining his position in the profession, Mr. Clark says (page 164), "In a metropolis like this, where the division of labor, while it cannot injure the individual, is attended with advantage to the public, the art of the dentist admits of several subdivisions. Presuming on this, the author has long restricted himself to one department, viz., Preserving the Natural Teeth, and with a degree of success fully commensurate with his expectations. These operations consist in scaling, that is, in freeing the teeth from extraneous matter, and Brushing spongy gums into a healthy state; in examining from time to time, teeth that from their shape and situation, are liable to decay, and at the proper time Cutting out the commencement of caries, and thereby Preventing its farther progress by the introduction of gold stoppings, by means of which a smooth even surface, incapable of retaining moisture long enough to rot in, is obtained, instead of the indentations where the caries commenced; in Curing tooth-ache and tender teeth; . . . relieving children from tooth-ache, to prevent the premature extraction of shedding teeth; and after the teeth have been lost through accident, heedless extraction, or old age, in pointing out the most appropriate mode of supplying their place by artificial means, together with the person most likely to do it well."

Mr. Clark lays particular stress upon the importance of preserving in effective usefulness the back teeth, and pointedly calls attention to the resulting ill effects not only of their loss, but also of their lost effectiveness when from caries or wear they become so shortened as to permit the anterior teeth to occlude too forcibly. In all such cases he recommends that the shortened teeth be covered with gold caps fitting the teeth, "as gloves fit the hands," building them up by soldering upon the masticating surfaces layer after layer of gold plate, or where much addition is required he prefers to rivet a block of ivory to the gold cap, carving it to represent the missing portion of the tooth. He insists that these caps, whether of gold or ivory, shall not be left smooth and flat on the mastication surfaces, but that they be carved to represent the hills and hollows of a natural tooth, and so conform to the occluding teeth that when the mouth is shut they "lock into each other as they formerly did into the surfaces of the natural teeth." Intervening spaces between capped teeth he recommends to be filled with properly carved blocks of ivory riveted to gold plate fitted to the gums between the teeth and made continuous with the gold caps. He recommends that those portions of the caps visible from the front be cut away, and remarks of one successful case, "no trace of gold or artificial teeth could be observed on a casual glance at the ladies' mouth." He gives in detail a number of cases successfully treated by dentures that must have closely resembled the removable bridges of the present day, and claims for the method suggested that not only are the anterior teeth relieved from undue pressure so that teeth loosened from that cause have regained their former firmness, but, as the pressure of mastication is borne by the capped teeth, the gums being thus relieved, the dentures are worn with much more comfort and satisfaction.

The first case Mr. Clark cites he gives as the date when treatment was commenced "about the middle of the year 1828," and in a later work published at London, 1836 ("Teeth and Dentism"), most of his remarks upon this subject are repeated.

It may be well to state that these methods are not given by Mr. Clark as originating with himself. From the language used I infer that they were methods of which he approved, well known to the dental art. While bridges supported in various ways by pins, dowels, or collars are known to be very old, and are illustrated in works I have of an earlier date ("L'Art du Dentiste," par L. Laforgue, Paris, 1802; "Traité complet de l'Art du Dentiste," par F. Maury, Paris, 1828, and others), these are the only references to the early use of gold caps for protecting carious teeth and supporting dentures I have so far met with, probably a more careful and extended search might bring others to light. Jobson ("A Treatise on the Anatomy and Physiology of the Teeth," by David Wemyss Jobson, M.R.C.S.E., reprint, Baltimore, 1844) refers to their use in connection with plates and obturators, pages 112 and 118, paragraphs 121 and 122.

